# 

**Published:** 1884-07

**Authors:** 


### CONTENTS:

| | PAGE. |
|---|---|
| ORIGINAL COMMUNICATIONS: | |
| Epilepsy. By Floyd S. Crego, M. D. | 531 |
| Case of Fracture of the Skull. By Chas A. Wall, M. S., M. D. | 543 |
| SOCIETY REPORTS: | |
| Erie County Medical Society, | 546 |
| SELECTIONS: | |
| The Influence of Worry and Nervous Shock on the Uterus and its Functions, | 554 |
| Meat, | 558 |
| Metallic Impurities in Canned Meats, | 563 |
| CORRESPONDENCE: | |
| Letter from Dr. H. R. Hopkins, | 560 |
| EDITORIAL: | |
| American Medical Association, | 565 |
| Niagara University, | 567 |
| EDITORIAL NOTES: | |
| Editorial Notes, | 567 |
| REVIEWS: | |
| Elementary Principles of Electro-Therapeutics. By C. M. Haynes, M. D | 568 |
| A Practical Treatise on Surgical Diagnosis. By Ambrose L. Ranney, M. D. | 569 |
| Auscultation, Percussion and Urinalysis. By C. Henri Leonard, M. A., M. D. | 570 |

